# Abacavir Increases Purinergic P2X7 Receptor Activation by ATP: Does a Pro-inflammatory Synergism Underlie Its Cardiovascular Toxicity?

**DOI:** 10.3389/fphar.2021.613449

**Published:** 2021-03-31

**Authors:** Víctor Collado-Díaz, Maria Ángeles Martinez-Cuesta, Maria Amparo Blanch-Ruiz, Ainhoa Sánchez-López, Patricia García-Martínez, José E Peris, Iris Usach, Maria Dolores Ivorra, Alessandra Lacetera, Sonsoles Martín-Santamaría, Juan V. Esplugues, Angeles Alvarez

**Affiliations:** ^1^ Departamento de Farmacología, Facultad de Medicina, Universidad de Valencia, Valencia, Spain; ^2^ CIBERehd, Valencia, Spain; ^3^ Departamento de Tecnología Farmacéutica, Facultad de Farmacia, Universidad de Valencia, Valencia, Spain; ^4^ Centro de Investigaciones Biológicas Margarita Salas, Consejo Superior de Investigaciones Científicas (CSIC), Madrid, Spain; ^5^ FISABIO- Fundación Hospital Universitario Dr. Peset, Valencia, Spain

**Keywords:** abacavir, adenosine triphosphate, P2X7 receptor, leukocyte-endothelium interactions, cardiovascular diseases, allosteric modulator

## Abstract

The cardiovascular toxicity of Abacavir is related to its purinergic structure. Purinergic P2X7-receptors (P2X7R), characterized by activation by high concentrations of ATP and with high plasticity, seem implicated. We appraise the nature of the interplay between Abacavir and P2X7R in generating vascular inflammation. The effects of Abacavir on leukocyte-endothelium interactions were compared with those of its metabolite carbovir triphosphate (CBV-TP) or ATP in the presence of apyrase (ATP-ase) or A804598 (P2X7R-antagonist). CBV-TP and ATP levels were evaluated by HPLC, while binding of Abacavir, CBV-TP and ATP to P2X7R was assessed by radioligand and docking studies. Hypersensitivity studies explored a potential allosteric action of Abacavir. Clinical concentrations of Abacavir (20 µmol/L) induced leukocyte-endothelial cell interactions by specifically activating P2X7R, but the drug did not show affinity for the P2X7R ATP-binding site (site 1). CBV-TP levels were undetectable in Abacavir-treated cells, while those of ATP were unaltered. The effects of Abacavir were Apyrase-dependent, implying dependence on endogenous ATP. Exogenous ATP induced a profile of proinflammatory actions similar to Abacavir, but was not entirely P2X7R-dependent. Docking calculations suggested ATP-binding to sites 1 and 2, and Abacavir-binding only to allosteric site 2. A combination of concentrations of Abacavir (1 µmol/L) and ATP (0.1 µmol/L) that had no effect when administered separately induced leukocyte-endothelium interactions mediated by P2X7R and involving Connexin43 channels. Therefore, Abacavir acts as a positive allosteric modulator of P2X7R, turning low concentrations of endogenous ATP themselves incapable of stimulating P2X7R into a functional proinflammatory agonist of the receptor.

## Introduction

Abacavir (ABC), a guanosine analogue belonging to the Nucleoside Reverse transcriptase Inhibitor (NRTI) family, has been widely prescribed to treat human immunodeficiency virus (HIV). However, it has been associated with a higher risk of myocardial infarction (Alvarez et al., 2017; Sabin et al., 2018), and so major clinical guidelines do not recommend the drug, particularly in patients with cardiovascular risk (Gunthard et al., 2016; Saag et al., 2018). There is growing evidence that this deleterious profile of actions involves the induction of a vascular inflammatory response; for instance, clinical concentrations of ABC have been shown to provoke human leukocyte-endothelial cell interactions (De Pablo et al., 2010; De Pablo et al., 2012), to promote the adherence of platelets to the endothelium (Alvarez et al., 2017), and to favor arterial thrombus formation in an animal model (Collado-Diaz et al., 2018).

**FIGURE 1:**
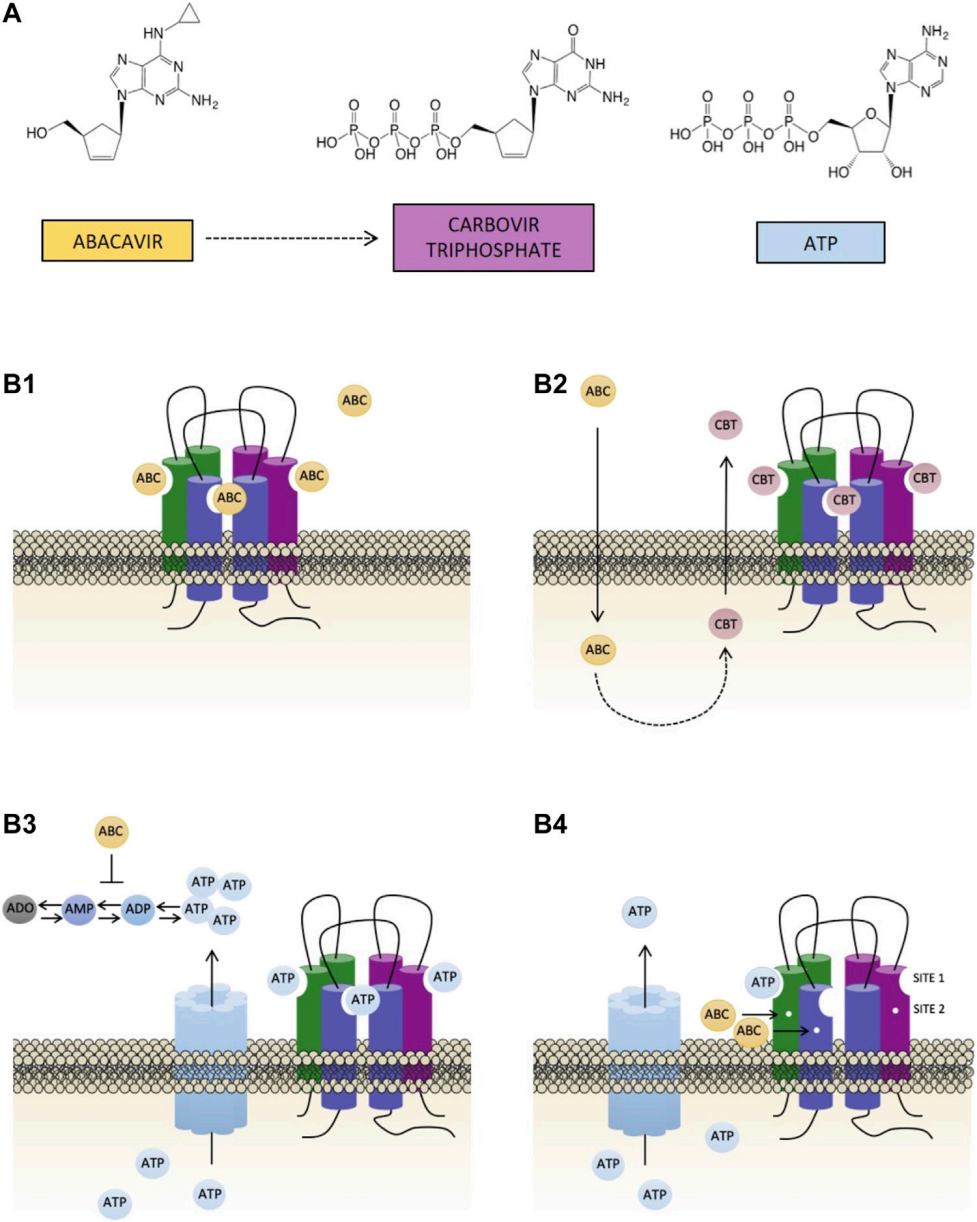
Chemical structures of abacavir, carbovir triphosphate and ATP, and potential causes of the activation of P2X7R. **(A)** Abacavir (ABC), its active metabolite carbovir triphosphate (CBV-TP), and ATP share a close structural similarity. **(B)** The bottom part of the figure represents the four potential scenarios of the interaction of ABC with the P2X7R evaluated in the present study: **(B1)** direct activation of the P2X7R by ABC through its binding to ATP-binding sites; **(B2)** ABC is metabolized into its active metabolite CBV-TP, which subsequently stimulates the P2X7R by binding to ATP-binding sites; **(B3)** ABC indirectly stimulates the P2X7R, and ATP is the molecule that binds to its binding sites. ABC could induce an enhancement in the levels of extracellular ATP either by interfering with the enzymes implicated in its synthesis/degradation or by mobilizing intracellular ATP to the extracellular space; and **(B4)** ABC allosterically modulates the activation of P2X7R. ABC binds to site 2, rendering this receptor more susceptible to direct activation by endogenous ATP.

The chemical structure of ABC - and even more so that of its active metabolite carbovir triphosphate (CBV-TP) (Barbarino et al., 2014) - is very similar to that of adenosine triphosphate (ATP) (Figure 1A), a major paracrine signaling molecule that is also capable of triggering a wide range of pro-inflammatory and pro-thrombotic events (Burnstock and Ralevic, 2013), by activating purinergic type 2 receptors (ionotropic P2X and metabotropic P2Y receptors). The mechanism responsible for the actions of ABC seems to be related to this purinergic component (De Pablo et al., 2010; Alvarez et al., 2017; Collado-Diaz et al., 2018) and involves a specific activation of purinergic ATP-P2X7 receptors (P2X7R) and endogenous ATP (Esplugues et al., 2016) - as its effects were specifically reverted by P2X7R antagonists but not by other P2XR or P2YR antagonists - in a convoluted interaction that is yet to be characterized.

P2X7R is widely expressed in immune system cells (macrophages, monocytes, neutrophils and mast cells) (Falzoni et al., 1995; Wilson et al., 2007; Burnstock and Boeynaems, 2014; Karasawa and Kawate, 2016; Karmakar et al., 2016) and has been repeatedly related to inflammatory conditions (Bours et al., 2011; Adinolfi et al., 2018). In addition, there is growing awareness of its relevance in cardiovascular diseases (Chen et al., 2018). Like all P2X receptors, it is an ATP-gated ion channel receptor with a trimeric structure (Rassendren et al., 1997; Moura et al., 2015); however, in contrast to other members of its family, P2X7R displays a high degree of plasticity with an allosteric binding pocket (Coddou et al., 2011; Moura et al., 2015; Rissiek et al., 2015; Di Virgilio et al., 2018), and requires unusually high extracellular concentrations of ATP (≥100 µmol/L) to become activated (Yan et al., 2010).

The aim of the present study was to characterize the interaction of ABC with the P2X7R. To do this we explored four potential scenarios (displayed graphically in Figure 1): 1) direct activation of P2X7R by ABC (Figure 1B1); 2) activation of P2X7R by the ABC’s active metabolite CBV-TP (Figure 1B2); 3) direct activation of P2X7R by ATP following the surge in ATP’s extracellular levels triggered by ABC (Figure 1B3); and 4) allosteric modulation of P2X7R by ABC, which would render this receptor more susceptible to direct activation by endogenous ATP (Figure 1B4). To put these hypotheses to the test we employed a series of functional and biochemical approaches in which we used Bz-ATP - a potent pharmacological agonist of P2X7R - as a positive control. We first compared the effects of ABC, CBV-TP and ATP on 1) human leukocyte-endothelium interactions, a hallmark of vascular inflammation; and 2) the expression of the leukocyte integrin Mac-1 (formed by the subunits CD11b and CD18), the adhesion molecule responsible for the binding of leukocytes to endothelial immunoglobulin ICAM-1 and their subsequent recruitment when in the presence of ABC (De Pablo et al., 2010; De Pablo et al., 2013). Thereafter, we quantified the effect of ABC on CBV-TP and ATP levels and evaluated the implication of various channels in order to determine if they are compatible with the actions of ABC and/or the functionality of the receptor. As ATP is labile and difficult to detect, we also analyzed the actions of ABC on ATP metabolism. We subsequently evaluated the ability of ABC, CBV-TP and ATP to bind to the P2X7R. Finally, we explored whether ABC acts allosterically by performing hypersensitivity studies with low concentrations of ABC and ATP.

## Methods

### Reagents

Physiological serum (190/12606059/1013) (Braun, Spain); Dulbecco’s PBS+ (DPBS+) (BE17-513F), Dulbecco’s PBS- (DPBS-) (17-512F), EGM-2 culture media (CC-3156) and fetal bovine serum (F7524) (Lonza, Spain); human serum albumin (A9080) (HSA, albuminate 25%), RPMI1640 (21875-034) supplemented with 20 mmol/L HEPES (15630-056), HBSS (H6648), fibronectin (F2006), dextran (31392), A804598 (SML0617), Suramin (S2671), Tris HCL (T1503), APCP (M8386) Polymyxin B (5291) and ATP (A26209) (Sigma Chemical Co., Spain); Ficoll-Paque TM Plus (17-1440-03) (GE Healthcare, Spain); PBS (20012-019), collagenase (17100-017) and trypsin (25200-072) (Gibco, Invitrogen, Spain); ABC (SRP00201a) (Sequoia Research Products, United Kingdom); CBV-TP (M1867) and [3H]A-804598 (MT1002211) (Moravek inc., USA); Ε-ATP (E004–05), E-ADP (E007-05), E-AMP (E005-10) and E-Ado (E011-10) (Biolog, Germany); Benzoylbenzoyl-ATP (Bz-ATP) (3312), Carbenexolone (3096), Probenecid (4107), Gap19 (5353) and Panx10 (3348) (Tocris Bioscience, United Kingdom); phycoerythrin [PE] mouse anti-human CD11b/Mac-1 (555388), fluorescein isothiocyanate [FITC] mouse anti-human CD18 (555923) and FITC mouse anti-human CD39 (561444) (BD PharmingenTM, Spain), PE mouse anti-human CD73 antibody [AD2] (ab157335) (abcam, United Kingdom) and FLUO-4 AM (F14201) (Life Technologies, USA).

### Human Samples

Human umbilical vein endothelial cells (HUVECs, passage 1) were harvested from fresh umbilical cords by collagenase treatment (De Pablo et al., 2010). Human peripheral blood polymorphonuclear (PMN) leukocytes were isolated from whole blood anticoagulated with sodium citrate drawn from healthy volunteers. HUVEC and PMNs (for adhesion assays, 4 h at 37°C), whole blood (for the analysis of Mac-1 expression, 4 h at 37°C) and PMNs (for calcium mobilization, 1 h at 37°C) were treated with clinically relevant concentrations (Harris et al., 2002; Moyle et al., 2009; Novitskaya et al., 2016) of ABC (20 μmol/L), ATP (0.05–10 μmol/L), CBV-TP (0.1–50 μmol/L), Bz-ATP (100 μmol/L), or vehicle. The concentrations of ABC employed (20 µM) that equals to 5.6 µg/mL of ABC tried to mimic plasma concentrations reached in patients (2–10 µg/mL) (Harris et al., 2002). In some cases cells were pretreated with: 1) antagonists for the ATP receptors: non-selective P2 (Suramin, 100 μmol/L, 60 min) or P2X7 (A804598, 1 μmol/L, 30 min); 2) an ATP-ase (apyrase, 1 IU/mL, 30 min) (Esplugues et al., 2016); 3) blockers of ATP-channels implicated in the activity of the P2X7R (Figure 1B3) 4) non-selective connexin and pannexin channels: Carbenexolone (CBX, 100 μmol/L, 30 min) and Probenecid (Prob, 150 μmol/L, 30 min); a mimetic inhibitory peptide that specifically blocks pannexin-1 gap junctions (Panx10, 300 μmol/L, 30 min); and selective connexin 43 hemichannel, which has no effect on gap junctions coupled with Gap19 (300 μmol/L, 30 min) (Abudara et al., 2014; Albalawi et al., 2017).

### Leukocyte-Endothelial Cell Interactions Under Flow Conditions

The parallel plate flow chamber system was employed, as previously described (Esplugues et al., 2016). In brief, coverslips containing confluent HUVEC monolayers were inserted in the chamber (37°C) and a portion (5 × 25 mm) was exposed to the flow. The chamber was mounted on an inverted microscope (Nikon Eclipse TE 2000-S, x40; Amstleveen, the Nederlands) connected to a videocamera (Sony Exware HAD; Koeln, Germany). PMNs were re-suspended in flow buffer [Dulbecco’s PBS, with (DPBS+) and without (DPBS-) Ca^2+^ and Mg^2+^ containing 20 mmol/L HEPES and 0.1% HSA] at 1 × 10^6^ cells/mL and drawn across the HUVEC monolayer at a controlled flow rate of 0.36 mL/min (estimated shear stress of 0.7 dyne/cm^2^). Single-field images were recorded over a 5-min period. Leukocyte rolling flux was calculated by counting the number of cells rolling across 100 μm^2^ of the monolayer during a 1-min period. Velocities of 20 consecutive leukocytes in the field of focus were analyzed by measuring the time required to travel 100 μm. Leukocyte adhesion was quantified after 5 min of leukocyte perfusion by analyzing 5–10 high power (40x) fields. Leukocytes were considered to be adherent after 30 s of stable contact with the monolayer (Esplugues et al., 2016).

### Expression of the Leukocyte Adhesion Molecule Mac-1

Blood samples were incubated with saturating amounts of antibodies against CD11b and CD18 (20 min, 4°C, in darkness), after which they were fixed and analyzed by a flow cytometer (FACScalibur Flow Cytometer, BD, Madrid, Spain) (De Pablo et al., 2010; De Pablo et al., 2012). Surface antigen expression (FITC or PE) was examined in neutrophils, which were identified by their specific features of size (forward-angle light scatter [FS]) and granularity (side-angle light scatter [SS]). Median fluorescence intensity was employed as a marker of the expression of the respective epitope, and fluorescence values were expressed as a percentage of the mean of the median fluorescence intensities vs. control cells.

### Calcium Mobilization in Polymorphonuclear Cells

PMNs were incubated with saturating concentrations of FLUO-4 AM (1h, 37°C, in darkness) concomitantly with drug treatments. Subsequently, they were fixed and analyzed by a flow cytometer (Papaioannou et al., 2016). PMNs were identified by their specific features of size (FS) and granularity (SS) and the median fluorescence intensity was employed as a marker of the FLUO-4 intensity, and fluorescence values were expressed as percentage of mean of the median fluorescence intensities vs. control cells.

### Quantification of Extracellular Levels of Nucleotides (Adenosine Triphosphate, Adenosine Diphosphate, Adenosine Monophosphate and Adenosine) and Carbovir Triphosphate

Supernatants of HUVEC and PMNs previously incubated with ABC (20 µmol/L, 37°C) were collected (3 and 8 min and 4 h), mixed with MeOH (10 min, 4°C) and centrifuged (10 min, 15.000 rpm). 100 µL of the respective samples were dried under N_2_ flow prior to the addition of H_2_O and subsequent analysis of the extracellular nucleotides by means of a 1200 Series uHPLC coupled to a 6495 QqQ/MS (Agilent Technologies) (Chen et al., 2009; Zhang et al., 2014).

To determine the levels of CBV-TP, supernatants of HUVEC and PMNs previously incubated with ABC (20 µmol/L, 37°C) were collected (at 4 h) and treated with phosphatase to degrade the CBV-TP to CBV, and CBV-TP was measured as total CBV (CBV-TP + CBV) by uHPLC coupled to a 6495 QqQ/MS (Agilent Technologies).

### Actions of Abacavir on Adenosine Triphosphate Metabolism

ATP is rapidly hydrolyzed by plasma membrane ectonucleotidases; CD39 (NTPDase-1) dephosphorylates ATP into ADP/AMP and CD73 (ecto-5′-NT) hydrolyzes AMP into adenosine (Figure 1B3). The effects of ABC on the protein expression and enzymatic activity of these enzymes were analyzed by flow cytometry and liquid chromatography, respectively. For flow cytometry, HUVEC or whole blood treated (4 h) with ABC (10–30 µmol/L) were incubated with saturating amounts of antibodies against CD39 and CD73 (20 min, 4°C, in darkness) (Borsellino et al., 2007), after which they were fixed and analyzed by a flow cytometer (FACScalibur Flow Cytometer, BD, Madrid, Spain) in a protocol similar to point “*Expression of the Leukocyte Adhesion Molecule Mac-1*.”

Cell-surface enzymatic activity was assessed by employing etheno-derivates - 1,N6-Etheno-adenosin-5′-triphosphate (E-ATP), 1,N6-Etheno-adenosin-5′-diphosphate (E-ADP), 1,N6-Etheno-adenosin-5′-monophosphate (E-AMP) and 1,N6-Etheno-adenosin (E-ADO)–which are degraded by the same enzymes as endogenous nucleotides (Synnestvedt et al., 2002). HUVEC or PMN were treated with ABC (20 μmol/L, 4 h), after which either E-ATP (5 μmol/L) or E-AMP (5 μmol/L) was added to the media. Next, the supernatant was collected (0, 1, 3, 10, 30 and 60 min), acidified to pH 3.5 with HCl and then spun (10,000 g for 20 s, 4°C), filtered (0.45 μm) and frozen (–80°C) until analysis via HPLC [(HP series 1050 quaternary pump and HP 1046A programmable fluorescence detector set at 300 nm (excitation) and 415 nm (emission)]. Chromatographic separation was performed on a Spherisorb ODS2 column (5 µm, 4.6 mm × 250 mm) at room temperature. The mobile phase consisted in a mixture of acetonitrile/methanol/buffer (12/8/80, v/v), being the buffer an aqueous solution of tetrabutylammonium hydrogen sulfate (20 mmol/L), potassium dihydrogen phosphate (25 mmol/L) and citric acid (10 mmol/L) with pH adjusted to 6.0. The flow rate was 1 mL/min and the injection volume was 25 µL. E-ATP and E-AMP assays permitted analysis of the production of E-ADP and E-ADO, respectively. CD39 activity was expressed as a percentage of E-ATP converted to E-ADP within the corresponding time frame, while that of CD73 was expressed as a percentage of E-AMP converted to E-ADO (Mattig et al., 2003; Falzoni et al., 2013).

### Effects of Apyrase on Abacavir Degradation

PMNs were treated with ABC and ATP (20 μmol/L) before being incubated with apyrase (1UI/mL). Levels of ABC and ATP were subsequently quantified by HPLC (0, 1, 3, 10, 60 and 240 min) (Esplugues et al., 2016). The HPLC equipment consisted of a quaternary pump SpectraSYSTEM P4000, an autosampler SpectraSYSTEM AS3000 and a spectrophotometric detector SpectraSYSTEM UV 6000LP set at 254 nm. Data was processed through “Chromquest chromatography workstation software Version 1.63”. The column was a Waters “Nova-Pack” C-18, the injection volume was 25 µL and the flow rate was 1 mL/min. The mobile phases and detection wavelengths were: acetonitrile/50 mmol/L sodium dihydrogen phosphate (10/90, v/v) and 285 nm in the case of ABC, and acetonitrile/20 mmol/L tetrabutylammonium hydrogen sulfate buffer (20/90, v/v) and 254 nm in the case of ATP.

### Studies of Binding to the Purinergic ATP-P2X7 Receptor

The ability of ABC, CBV-TP and ATP to bind to the P2X7R was evaluated by two approaches: 1) Radioligand receptor-binding assays, which assess the capacity of a ligand to shift the radioligand from the receptor; and 2) docking calculations, that predict different binding poses to the target and their binding energies.

#### Radioligand Receptor Binding Assays

Competition-binding assays (in duplicate) were carried out with a purified membrane preparation of HUVEC (Donnelly-Roberts et al., 2009). Cell membranes (75μg protein) were incubated (4°C, 60min) in 250μL of assay buffer (50mmol/L Tris-HCl, 0.1% BSA, pH 7.4) with 2.5nmol/L of a P2X7R antagonist ([3H]A-804598, 36.2Ci/mmol) in the absence or presence of increasing concentrations of unlabeled competitors [Bz-ATP, ATP, CBV-TP and ABC; 0.1µmol/L−1mmol/L]. The pharmacological ligand of the receptor Bz-ATP was employed as a positive control. Experiments were finalized with rapid vacuum filtration through Whatman GF/C fiberglass filters (GE Healthcare Europe GmbH, Barcelona, Spain) presoaked in 0.3% polyethyleneimine and using a Brandel cell harvester (M24R, Valley Research Iberica, Madrid, Spain). Filters were then washed five times with 3mL of ice-cold 50mmol/L Tris-HCl buffer (pH 7.4), after which the filter-bound radioactivity was measured (Packard Tri-Carb Liquid Scintillation Counter, PerkinElmer, Waltham, MA, USA). Non-specific binding was observed in the presence of 1mmol/L of unlabeled Bz-ATP. Specific binding is defined as total binding minus non-specific binding. Competition-binding data were fitted to a sigmoid curve by non-linear regression using GraphPad Prism 8.0 in order to obtain pIC50 values (-log of the concentration of the compound needed to inhibit 50% radio-ligand binding).

#### Purinergic ATP-P2X7 Receptor Docking

We assessed the docking of ligands (Bz-ATP, ATP, CBV-TP, ABC and A804598) to P2X7R. Regarding the 3D structure of the macromolecule, a homology model was derived with Swiss Model (https://swissmodel.expasy.org) by using the structure of panda P2X7R (PDB 5UsH) (Karasawa and Kawate, 2016), as template. The similarity between the *Homo sapiens* and Ailuropoda melanoleuca (panda) species was stated by sequence alignment employing the T-coffee server (http://tcoffee.crg.cat) obtaining a score of 97/100. Regarding the 3D structure of the ligands, it was built with Maestro suite (http://www.schrodinger.com/maestro) and their 3D geometry was optimized with the molecular mechanisms OPLS-2005 force field. AutoDockTools 1.5.6 program (http://autodock.scripps.edu/) was used to assign the Gasteiger-Marsili empirical atomic partial charges, and nonpolar hydrogens were merged. AutoDock 4.2 (http://autodock.scripps.edu) was used to dock the ligands towards to the P2X7R at the two different possible binding sites; one for ATP and another called the drug-binding pocket, to which A804598 binds (Karasawa and Kawate, 2016). For each site, a grid box was defined with dimensions of 50 Å × 70 Å x 60 Å and grid spacing of 0.375 Å. The box center was located at coordinates (x,y,z) of 171, 168, 142 and 162, 150, 283, for boxes at ATP and A804598 sites, respectively. The Lamarckian genetic algorithm (LGA) was used to find the appropriate binding conformations of ligands. The number of genetic algorithm runs was set to 200 poses to enhance the sampling. This allowed us to estimate a free binding energy (Kcal mol^−1^) for each one of the ligands. The complex (ligand-target) with the lowest predicted free binding energies are the most stable.

### Hypersensitivity Studies

The effects of increasing concentrations of ATP (0.001–20 µmol/L) on leukocyte rolling flux, expression of the Mac-1 subunit CD11b and calcium mobilization were evaluated in the presence of a concentration of ABC (1 µmol/L) that, by itself, has no effect on either parameter or, in some cases, in the presence of the positive allosteric modulator polymyxin B (8 μmol/L) (Ferrari et al., 2004; De Pablo et al., 2010).

### Ethics and Statistics

Umbilical cords and blood were obtained from Hospital Clínico Universitario (Valencia) whose Medical Ethical Committee approved the study, and all participating patients provided written informed consent. All the procedures performed complied with Spanish law. Data are presented as mean ± S.E.M. (*n* ≥ 4), and statistical analyses were performed (GraphPad Prism 8.0) by a person blinded to the experimental conditions. A one-way analysis of variance (ANOVA) with a Newman-Keuls post-hoc correction for multiple comparisons was employed, or a *t*-test where appropriate. A *p* value <0.05 was considered statistically significant.

## Results

### Comparison of the Pro-inflammatory Effects of Abacavir With Those of Adenosine Triphosphate and Carbovir Triphosphate and the Role of Purinergic Receptors in These Effects

**FIGURE 2:**
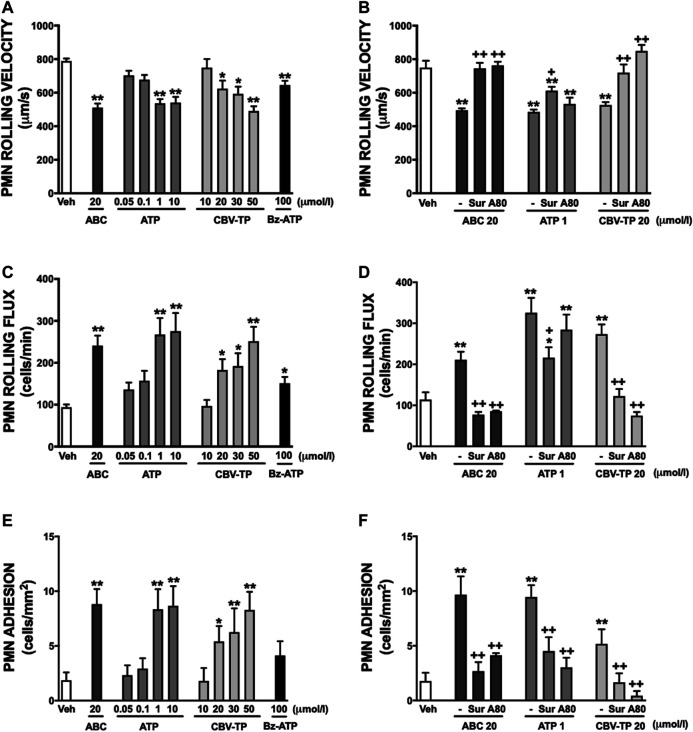
PMN-endothelial cell interactions. HUVEC and PMN were incubated (4 h) with ABC (20 µmol/L), ATP (0.05–10 µmol/L), CBV-TP (10–50 µmol/L), Bz-ATP (100 µmol/L) or vehicle (veh). In some cases, both cell types (HUVEC and PMN) were pretreated with suramin (Sur, non-selective P2R antagonist, 100 μmol/L, 60 min) or with A804598 (A80, P2X7R antagonist, 1 μmol/L, 30 min) prior to ABC (20 µmol/L), ATP (1 µmol/L) or CBV-TP (20 µmol/L). PMN rolling velocity **(A, B)**, PMN rolling flux **(C, D)** and PMN adhesion **(E, F)** were quantified. Results are mean ± SEM, Veh: *n* ≥ 14, ABC: *n* ≥ 7, ATP 0.05: *n* ≥ 4, ATP 0.1: *n* ≥ 13, ATP 1: *n* ≥ 6, ATP 10: *n* ≥ 6, CBV-TP 10: *n* ≥ 6, CBV-TP 20: *n* ≥ 5, CBV-TP 30: *n* ≥ 5, CBV-TP 50: *n* ≥ 6, Bz-ATP 100: *n* ≥ 6, ABC 20 + Sur: *n* ≥ 4, ABC 20 + A80: *n* ≥ 4, ATP 1 + Sur: *n* ≥ 6, ATP 1 + A80: *n* ≥ 5, CBV-TP 20 + Sur: *n* ≥ 5, CBV-TP 20 + A80: *n* ≥ 5. *p < 0.05 or **p < 0.01 vs. corresponding value in vehicle-treated group. +*p* < 0.05 or ++*p* < 0.01 vs. corresponding value in ABC, ATP or CBV-TP-treated group (ANOVA followed by Newman-Keuls test).

**FIGURE 3:**
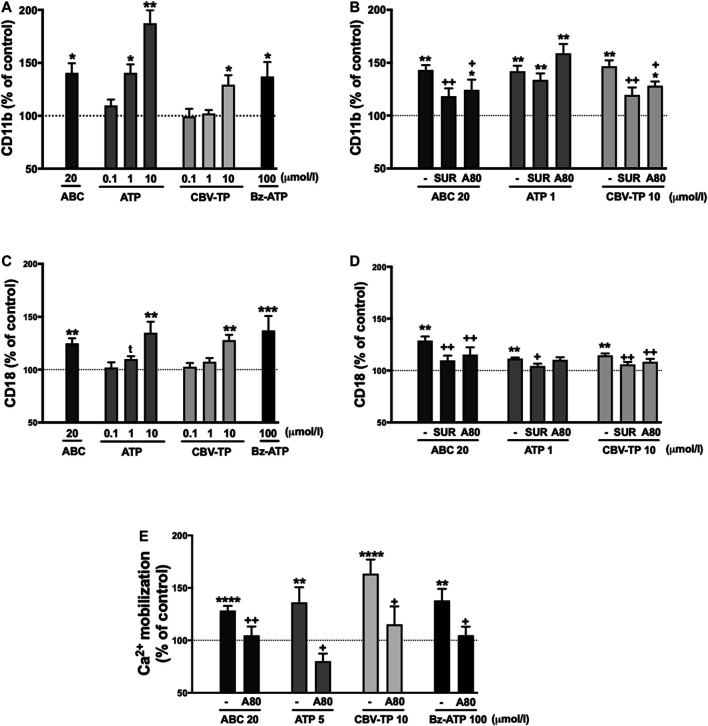
Mac-1 expression and calcium influx in neutrophils. Whole blood (4 h) for Mac-1 expression or PMN (1 h) for calcium influx were treated with ABC (20 µmol/L), ATP (0.1–10 µmol/L) or CBV-TP (0.1–10 µmol/L), and the surface expression on neutrophils of the two subunits of Mac-1 CD11b **(A, B)** and CD18 **(C, D)**, and calcium mobilization **(E)** were quantified. In some cases, blood or PMN were pretreated with suramin (Sur, nonselective P2R antagonist, 100 μmol/L, 60 min) or A804598 (A80, P2X7R antagonist, 1 μmol/L, 30 min) prior to treatment with ABC (20 µmol/L), ATP (1 µmol/L), ATP (5 µmol/L), CBV-TP (10 µmol/L) or Bz-ATP (100 µmol/L). Fluorescence values are expressed as percentage of mean of median fluorescence intensities of control cells (dotted line). Results are mean ± SEM, Veh: *n* ≥ 11, ABC 20: *n* ≥ 15, ATP 0.1: *n* ≥ 6, ATP 1: *n* ≥ 6, ATP 5: *n* = 10, ATP 10: *n* ≥ 7, CBV-TP 0.1: *n* ≥ 6, CBV-TP 1: *n* ≥ 10, CBV-TP 10: *n* ≥ 5, Bz-ATP 100: *n* ≥ 6, ABC 20 + Sur: *n* ≥ 9, ABC 20 + A80: *n* ≥ 5, ATP 1 + Sur: *n* ≥ 10, ATP 1 + A80: *n* ≥ 11, ATP 5 + A80: *n* ≥ 4, CBV-TP 10 + Sur: *n* ≥ 12, CBV-TP 10 + A80: *n* ≥ 4, Bz-ATP 100 + A80: *n* ≥ 4, *p < 0.05, **p < 0.01 or ****p < 0.0001 vs. corresponding value in vehicle-treated group. +*p* < 0.05 or ++*p* < 0.01 vs. corresponding value in ABC, ATP or CBV-TP-treated group (ANOVA followed by Newman-Keuls test), ^*t*^ *p*<0.05 vs. corresponding value in vehicle-treated group (*t*-test).

Clinical concentrations of ABC (20 µmol/L), similarly to Bz-ATP, exhibited a profile of pro-inflammatory actions that were in accordance with those demonstrated previously (De Pablo et al., 2010; De Pablo et al., 2012; Esplugues et al., 2016); in other words, they induced a significant decrease in human leukocyte rolling velocity (Figure 2A) while increasing leukocyte rolling flux (Figure 2C) and adhesion (Figure 2E) to endothelial cells. In PMN these effects were accompanied by a significant enhancement in the expression of the two subunits of the adhesion molecule Mac-1: CD11b (Figure 3A) and CD18 (Figure 3C). Both ATP and CBV-TP mimicked the effects of ABC, though these actions depended on the dose (Figures 2A,C,E, 3A,C); while CBV-TP induced PMN-endothelium interactions or overexpression of the two subunits of the β_2_-integrins in a range of concentrations similar to those of ABC (10–20 µmol/L), doses of ATP 10–20 times lower (1 µmol/L) were capable of producing the same effects.

These leukocyte-endothelial cell interactions were prevented by the non-selective P2 receptor antagonist Suramin. However, the specific P2X7R antagonist A804598 induced inconsistent results; although it reversed the actions of ABC and CBV-TP, it failed to modify those of ATP (Figures 2B,D). Similar results were obtained with respect to overexpression of CD11b and CD18. Suramin prevented the effects of the three potential ligands, while A804598 only reversed those of ABC and CBV-TP (Figures 3B,D). In this way, CBV-TP and ATP induced a profile of proinflammatory actions similar to those of ABC, but one which was not fully P2X7R-dependent in the case of ATP.

Additionally, all Bz-ATP, ABC, ATP and CBV-TP produced calcium influx in a P2X7R dependent manner (Figure 3E).

### Effects of Abacavir on Carbovir Triphosphate and Adenosine Triphosphate Levels, and on Adenosine Triphosphate Metabolism

**FIGURE 4:**
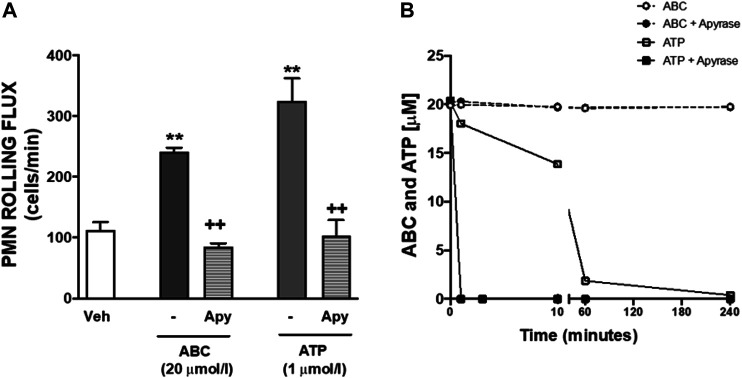
Effects of apyrase on PMN-endothelial cell interactions and on levels of ABC and ATP. **(A)** HUVEC and PMN were incubated (4 h) with ABC (20 µmol/L), ATP (1 µmol/L) or vehicle (veh). In some cases, both cell types (HUVEC and PMN) were pretreated with apyrase (Apy, ATPase, 100 μmol/L, 60 min) prior to ABC or ATP treatment. PMN rolling flux was quantified. **(B)** PMNs were incubated with ABC (20 µmol/L) or ATP (1 µmol/L). In some other experiments, cells were pretreated with apyrase (100 UI/L) and levels of ABC and ATP in the supernatants were quantified by HPLC (at 0, 1, 10, 60 and 240 min). Results are mean ± SEM, Veh: *n* = 15, ABC 20: *n* = 15, ABC 20 + Apy: *n* = 9, ATP 1: *n* = 6, ATP 1 + Apy: *n* = 4. **p < 0.01 vs. corresponding value in vehicle-treated group. ++*p* < 0.01 vs. corresponding value in ABC or ATP-treated group (ANOVA followed by Newman-Keuls test).

**FIGURE 5:**
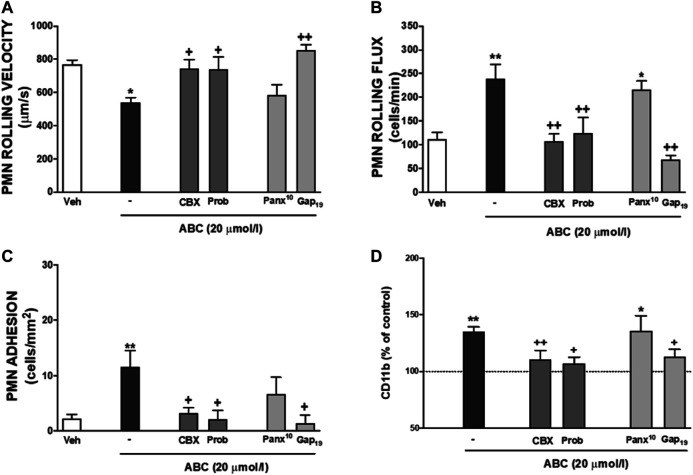
Role of endogenous ATP released by different channels on leukocyte-endothelial interactions induced by ABC. HUVEC and PMN or whole blood were incubated (4 h) with ABC (20 µmol/L). In some cases, they were pretreated with Carbenexolone (pannexin and connexin channels blocker, CBX, 100 µmol/L), Probenecid (Prob, pannexin and connexin channels and P2X7 receptor blocker, 150 µmol/L), Panx10 (mimetic inhibitory peptide that blocks pannexin-1 gap junctions, 300 µmol/L) or Gap_19_ (selective Connexin43 channel blocker Gap_19_, 300 µmol/L). PMN rolling velocity **(A)**, PMN rolling flux **(B)** and PMN adhesion **(C)** were quantified after assembling the flow chamber and the expression of one of the two subunits of Mac-1 CD11b **(D)** on neutrophils was quantified by flow cytometry. Fluorescence values are expressed as percentage of mean of median fluorescence intensities of control cells (dotted line). Results are mean ± SEM, Veh: *n* ≥ 7, ABC 20: *n* ≥ 6, ABC 20 + CBX: *n* ≥ 6, ABC 20 + Prob: *n* ≥ 4, ABC 20 + Pa*n*x^10^: *n* ≥ 5, ABC 20 + Gap_19_: *n* ≥ 4. *p < 0.05 or **p < 0.01 vs. corresponding value in vehicle-treated group. +*p* < 0.05 or ++*p* < 0.01 vs. corresponding value in ABC-treated group (ANOVA followed by Newman-Keuls test).

Treatment of HUVEC or PMNs with ABC (20 µmol/L, 3 min, 8 min, 4 h) did not generate detectable extracellular amounts of either CBV-TP or CBV in our system. Likewise, ABC (20 µmol/L; 3m, 8min, 4 h) failed to modify extracellular ATP levels in either cell type (Table 1, and unshown data). Subsequently, we explored whether ABC influenced the metabolism of ATP by evaluating the expression and activity of CD39 and CD73 in HUVEC and PMNs, and found that the compound did not modify either their protein expression (Supplementary Figure S1) or enzymatic activity (Supplementary Figure S2). Although these results suggest that ABC does not significantly increase extracellular levels of endogenous ATP, the fact that apyrase blocked the effects of ABC and ATP on PMN-rolling flux (Figure 4A) supports the idea that ABC relies on endogenous ATP to exert its actions, as this enzyme degraded ATP without modifying the levels of ABC (Figure 4B). Pulses of ATP from the intracellular compartment to the extracellular space through connexin (mostly located in leukocytes) and pannexin (panx1, more widely distributed) channels could also account for the interaction of ATP with binding sites of P2X7R (Willebrords et al., 2017; Zhou et al., 2019). The fact that the effects of ABC on leukocyte-endothelium interactions (Figures 5A–C) and expression of CD11b (Figure 5D) were prevented by two non-specific channel blockers like CBX and Prob and by the specific connexin channel blocker Gap19, but not by the specific pannexin channel blocker Panx10, suggest that connexin channels are essential for its vascular pro-inflammatory actions.

**TABLE 1:** Extracellular levels of nucleotides on neutrophils and on endothelial cells.

		Extracellular concentration of nucleotides (nmol/L)							
		ATP		ADP		AMP		ADO	
		3 min	8 min	3 min	8 min	3 min	8 min	3 min	8 min
PMN	Vehicle	59.5 ± 17.5	64.1 ± 16.8	60.7 ± 17.1	55.7 ± 20.3	253.9 ± 1.2	289.3 ± 34.5	38.2 ± 5.5	44.5 ± 6.7
	ABC (20 µmol/L)	51.5 ± 7.5	50.8 ± 7.6	47.9 ± 6.9	43.8 ± 0.3	224.9 ± 28.4	209.5 ± 50.5	34.6 ± 0.6	32.6 ± 7.8
HUVEC	Vehicle	84.5 ± 23.3	55.1 ± 12.6	32.5 ± 7.9	28.9 ± 2.9	9.9 ± 0.5	10.3 ± 1.3	15.5 ± 10.8	21.5 ± 4.1
	ABC (20 µmol/L)	66.8 ± 9.4	54.55 ± 7.5	25.1 ± 7.2	25.1 ± 2.8	10.4 ± 2.1	11.4 ± 0.3	19.3 ± 8.7	19.2 ± 11.3

PMN and HUVEC were treated with ABC (20 μmol/L) or vehicle, and the supernatant was collected for 3 and 8 min. Concentrations of ATP, ADP, AMP and Adenosine were quantified by HPLC. Results are mean ± SEM, PMN vehicle: n ≥ 4, PMN ABC (20 μmol/L): n ≥ 4, HUVEC vehicle: n ≥ 5, HUVEC ABC (20 μmol/L): n ≥ 5.

### Ligand-Purinergic ATP-P2X7 Receptor Binding and Docking Studies

**FIGURE 6:**
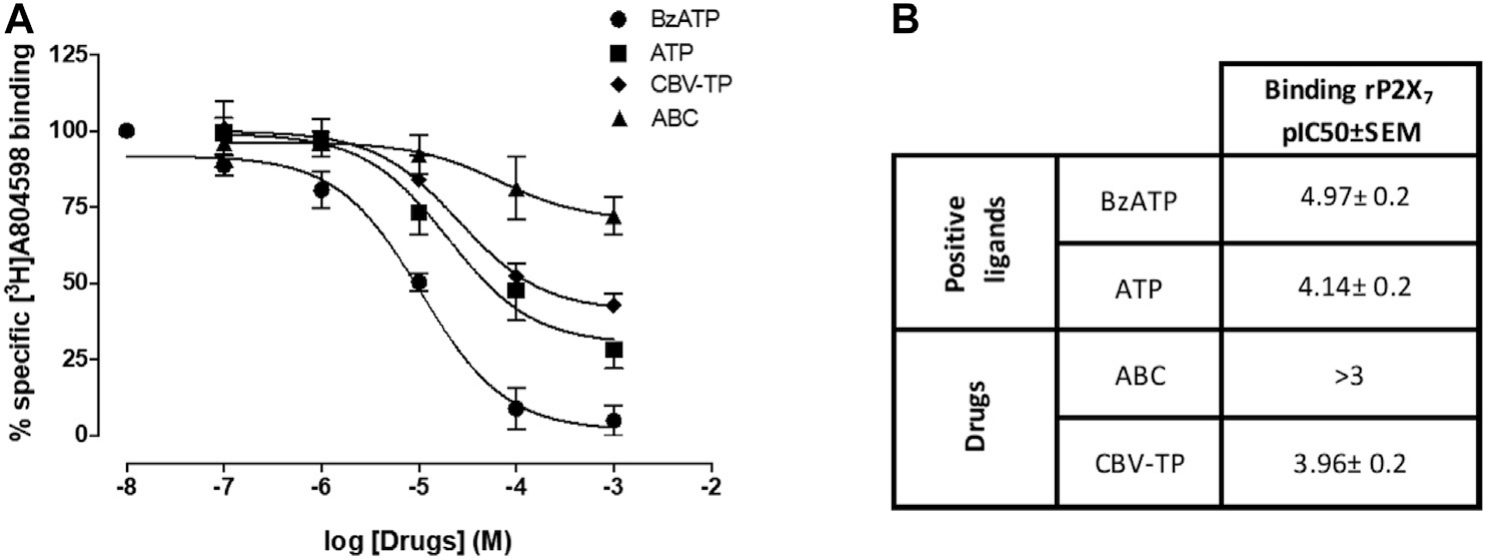
Studies of radioligand-binding to P2X7R. **(A)** Displacement of [3H]A-804598 (P2X7 receptor antagonist, 2.5 nmol/L) by increasing the concentrations of Bz-ATP, ATP, CBV-TP and ABC (0.1 µmol/L-1 mmol/L) at membrane preparations of HUVEC. Results are mean ± SEM, Bz-ATP: *n* ≥ 3, ATP: *n* ≥ 6, CBV-TP: *n* ≥ 6, ABC: *n* ≥ 5; each experiment was performed in duplicate. **(B)** Affinities (pIC50 values) of Bz-ATP, ATP, ABC and CBV-TP for the human P2X7R.

As expected, Bz-ATP (0.01µmol/L-1mmol/L) shifted (in a concentration-dependent manner) the binding of the radioligand to the P2X7R (pIC50 = 4.97 ± 0.2). ATP and CBV-TP also produced a shift, but with less effectiveness than Bz-ATP, while ABC failed to exert any influence in this respect, even at concentrations of 1 mmol/L (pIC50 < 3) (Figure 6). These results imply that ATP and CBV-TP have affinity for the P2X7R at the binding site of labeled A804598, while ABC does not.

**FIGURE 7:**
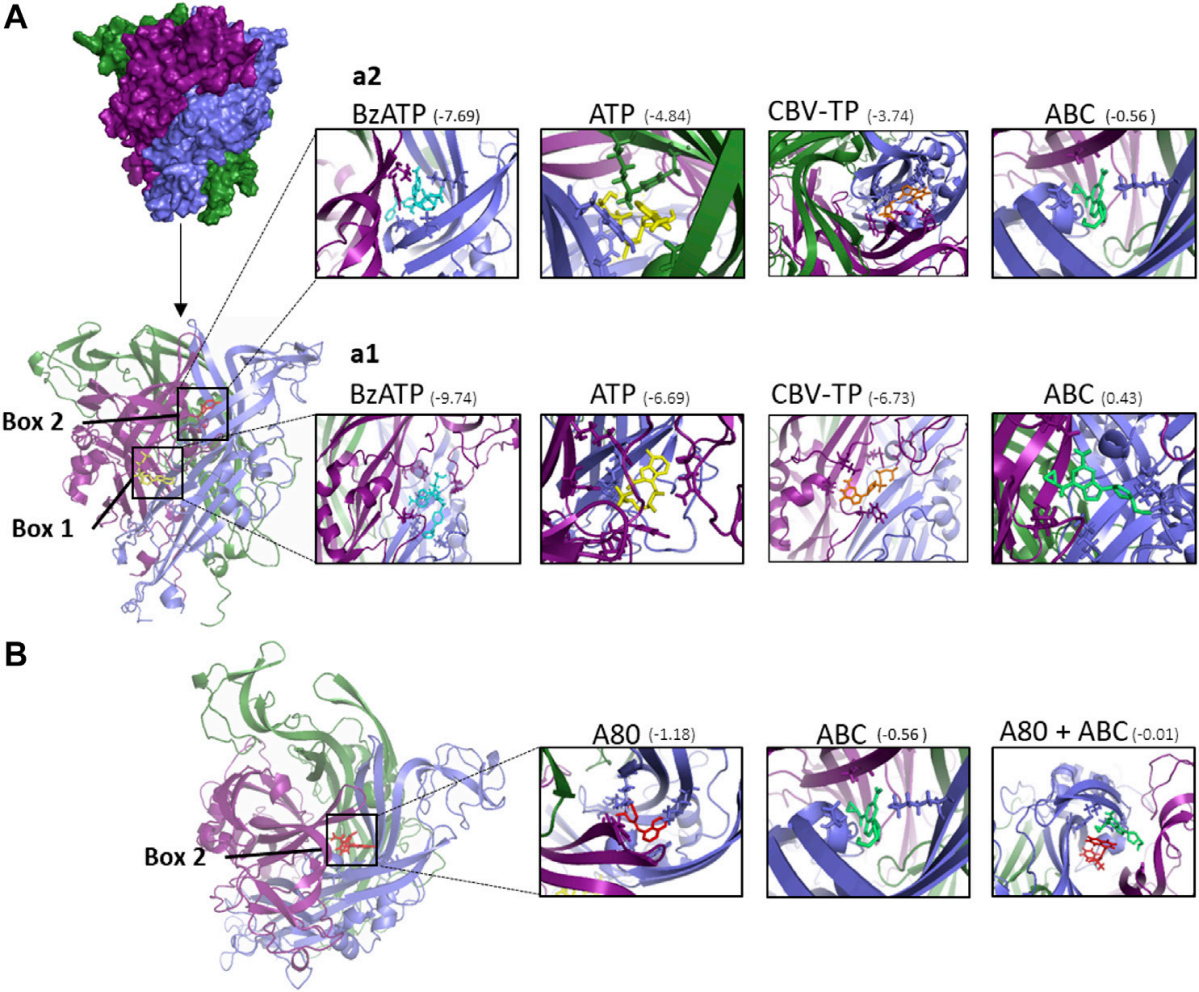
Docking calculations of ABC and other ligands to P2X7 receptor. **(A)** Structure of human P2X7R built by homology modeling based on the X-ray crystal structure of panda P2X7R (5U2H). The P2X7R trimer is represented as a molecular surface. The three monomers are colored in deep purple, slate blue, and forest green. Each monomer is shaped like a dolphin (Hattori and Gouaux, 2012). Docked poses at ATP binding site (box 1) and A804598 (A80) binding site (box 2) of the different compounds studied. The P2X7R trimer is represented as a cartoon. ATP is depicted in yellow, A804598 in red, Bz-ATP in blue and CBV-TP in orange and ABC in lime green. (**a1**) Details of the ligands docked at box 1 showing some amino acid side chains at the interaction site. (**a2**) Details of the ligands docked at box 2 showing some amino acid side chain at the interaction site. **(B)** Superimposition of docked poses of P2X7R antagonist A804598 (A80, in red) and ABC (in lime green) at the binding site two of the P2X7R. The predicted binding energies are indicated under parenthesis (kcal mol^−1^).

Possible binding sites and binding poses were explored for ligands Bz-ATP, ATP, CBV-TP, ABC and A804598 into P2X7R by means of docking calculations. Two binding sites of the P2X7R were explored: site 1 (named ATP site) and site 2 (named allosteric modulation site or A804598 site) (Figure 7A), as reported (Karasawa and Kawate, 2016). Bz-ATP presented the best predicted affinity for both sites, showing higher predicted affinity for site 1 than for site 2. ATP and CBV-TP were also capable of binding to both sites, though exhibiting higher affinity for site 1 than for site 2 (Figure 7A). For ABC, however, unfavorable binding energy was predicted at site 1 (0.43 Kcal mol^−1^) while low but favorable binding energy was predicted at site 2 (−0.56 Kcal mol^−1^) (Figure 7A). These results suggest that ABC could be acting as an allosteric modulator through the binding to the site 2 of P2X7R. Figure 7B shows that both the antagonist A804598 and ABC are capable of binding to different places within site 2, which is compatible with the observation (shown in Figure 6) that ABC did not displace the radioligand A804598 from its binding site.

### Effects of Abacavir as a Positive Allosteric Modulator of Purinergic ATP-P2X7 Receptor

**FIGURE 8:**
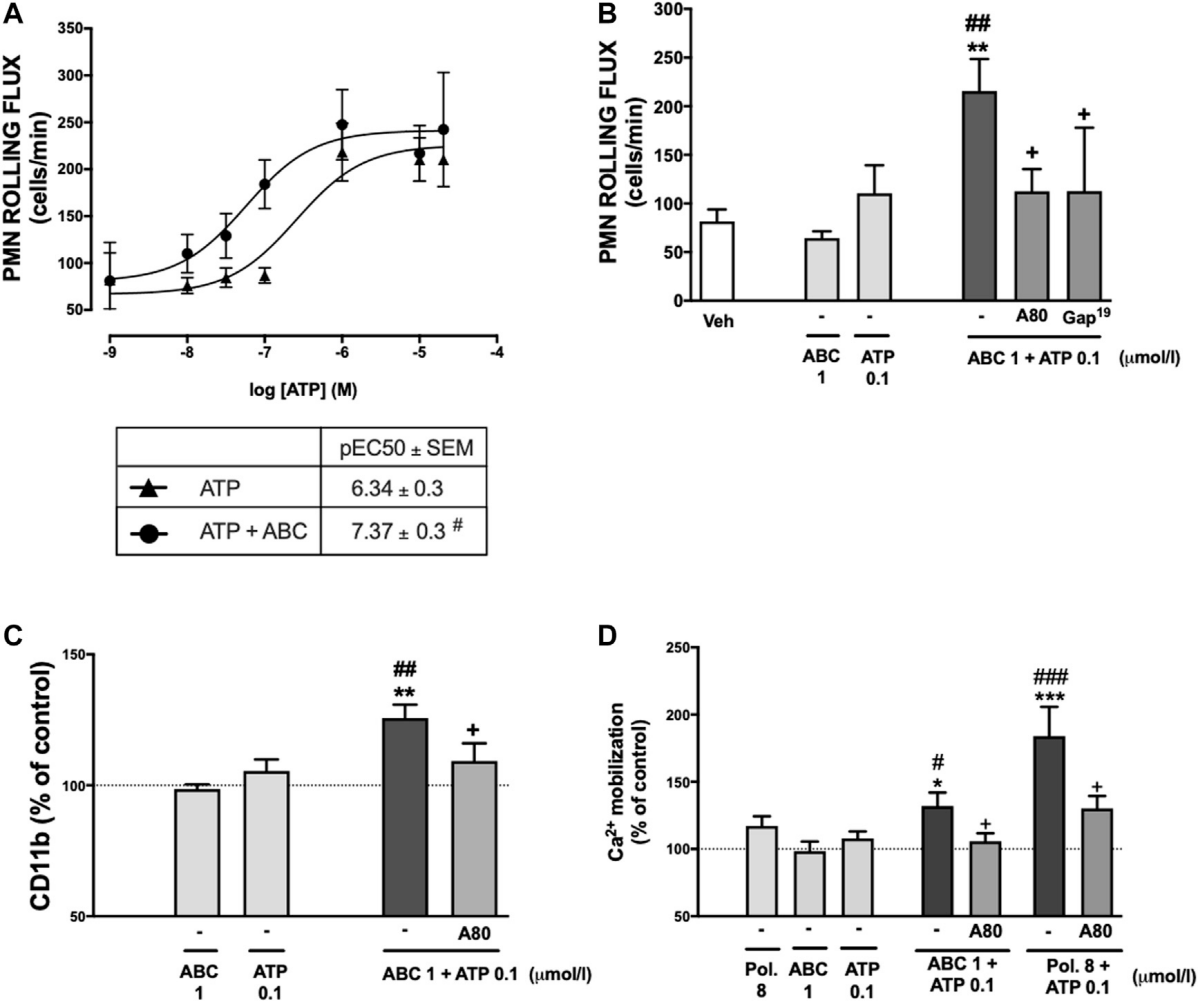
Potentiation by ABC of the effects of ATP on PMN-endothelial cell interactions, the expression of CD11b and on calcium influx. HUVEC and PMN were treated (4 h) with ATP (0.001–20 µmol/L) in the presence or absence of ABC (1 µmol/L) and PMN rolling flux **(A)** was analyzed. Doses of ABC (1 µmol/L) and ATP (0.1 µmol/L) were selected to treat individually or in combination both PMN and HUVEC or whole blood. In some cases, both HUVEC and PMN or whole blood were pretreated with A804598 (A80, P2X7R antagonist, 1 μmol/L, 30 min) prior to ABC (1 µmol/L) and ATP (0.1 µmol/L). PMN rolling flux **(B)** was quantified after assembling the flow chamber and the expression of one of the two subunits of Mac-1 CD11b **(C)** on neutrophils was quantified by flow cytometry. **(D)** PMNs were treated (1 h) with Polymyxin B (Pol. 8 µmol/L), ABC (1 µmol/L) and ATP (0.1 µmol/L) individually or in combination and, in somes cases, PMN were pretreated with A804598 (A80, P2X7R antagonist, 1 μmol/L, 30 min) prior to Polymyxin B (Pol. 8 µmol/L) and ATP (0.1 µmol/L) or ABC (1 µmol/L) and ATP (0.1 µmol/L). Calcium mobilization was quantified by flow cytometry. Fluorescence values are expressed as a percentage of mean fluorescence intensities of control cells (dotted line). Results are mean ± SEM, Veh: *n* ≥ 5, Pol. 8: *n* = 3, ABC 1: *n* ≥ 5, ATP 0.1: *n* ≥ 5, ABC 1 + ATP 0.1: *n* ≥ 5, ABC 1 + ATP 0.1 + A80: *n* ≥ 5, ABC 1 + ATP 0.1 + Gap^19^: *n* ≥ 3 Pol. 8 + ATP 0.1: *n* = 3, Pol. 8 + ATP 0.1 + A80: *n* ≥ 3. *p < 0.05, **p < 0.01 or ***p < 0.001 vs. corresponding value in ABC-treated group. #*p* < 0.05, ##*p* < 0.01 or ###*p* < 0.001 vs. corresponding value in ATP-treated group. +*p* < 0.05 or ++*p* < 0.01 vs. corresponding value in the ABC + ATP-treated group (ANOVA followed by Newman-Keuls test).

Concentration-response curves of the effect of ATP (0.001–20 µmol/L) on leukocyte rolling flux were constructed in the absence or presence of a concentration of ABC (1 µmol/L) that had no effect by itself on this pro-inflammatory parameter. The presence of ABC produced a significant increase in the potency of ATP, but not in its efficacy, to induce leukocyte rolling flux (Figure 8A). Thus, in the absence of ABC the pEC50 was 6.34 ± 0.3, while it was significantly lower (7.37 ± 0.3, *p* ≤ 0.05) in its presence. Likewise, the increase in PMN rolling flux, expression of CD11b and calcium influx induced by the combination of ABC and ATP (1 and 0.1 µmol/L, respectively; both of which concentrations were ineffective by themselves) was prevented by the specific P2X7R antagonist A804598 (Figures 8B–D), therefore implicating this receptor in said actions. As expected, the positive allosteric modulator of the P2X7R polymyxin B also potentiated calcium influx induced by low concentrations of ATP in a P2X7R manner, thus confirming the reliability of the functional assays. Finally, the involvement of P2X7R-associated connexin channels was made evident, as the effects of a combination of low doses of ABC + ATP on PMN rolling flux were reverted by Gap19 (Figure 8B).

## Discussion

This study analyzes the interaction between ABC and P2X7R that would appear to be responsible for the drug’s vascular inflammatory effects (Alvarez et al., 2017; Sabin et al., 2018; Sabin et al., 2016). The possibility that ABC binds to ATP-binding sites and directly activates P2X7R (Figure 1B1) was rapidly ruled out, as binding analysis demonstrated that ABC could not shift the radioligand from this receptor, thus indicating a lack of affinity. Likewise, docking studies were not definitive, as ABC clearly bonded to site 2, though with a binding energy noticeably lower than that of ATP. This was to be expected given ABC’s structure, which does not include the three phosphate chains so characteristic of ATP (Alvarez et al., 2017).

The profile of proinflammatory actions induced by CBV-TP in our experiments was similar to that of ABC; namely, induction of leukocyte-endothelium interactions, expression of the leukocyte adhesion Mac-1 and calcium influx, all of which were mediated by P2X7R. ABC is converted intracellularly into CBV-TP (Barbarino et al., 2014), subsequently transported extracellularly through pore-forming channels (Fortes et al., 2004; Eltzschig et al., 2006; Baranova et al., 2004), and activates P2X7R by interacting with ATP-binding sites (Figure 1B2). Binding and docking studies confirmed this hypothesis, showing the metabolite to act as a ligand of P2X7R with a similar affinity to ATP. However, although we detected increases in its intracellular levels (around 300 fmol/million cells in endothelial cells) (Alvarez et al., 2017), changes in its extracellular levels were not compatible with a profile of proinflammatory actions. This is in keeping with the low plasma levels of CVB-TP (in the range of 100 fmol/million cells) detected in HIV patients treated with ABC (Moyle et al., 2009) and is evidence against a role for this metabolite in the clinical vascular toxicity of ABC.

However, the fact that ABC’s effects on leukocyte-endothelium interplay were reverted by apyrase–an enzyme that hydrolyses ATP but not ABC, as we demonstrate here - highlights the importance of the role of endogenous ATP in ABC’s actions (Figure 1B3), something hinted at by previous findings (Esplugues et al., 2016). Radio-ligand and docking analysis confirmed ATP as the natural ligand of P2X7R, while low concentrations of exogenous ATP (1 µmol/L) reproduced the profile of proinflammatory actions of ABC. This led us to suspect that ABC-released ATP interacts with P2X7R, and thus may be responsible for the drug’s actions (Figure 1B3). Such an increase in the levels of extracellular ATP could be instigated by: 1) mobilization (by ABC) of intracellular ATP to the extracellular space through various channels; or 2) interference of ABC with the enzymes implicated in ATP synthesis or degradation. Our observation that blockade of connexin channels prevented the proinflammatory effects of ABC supports this idea of ATP mobilization from within the cell. Nonetheless, we were unable to detect a significant increase in the levels of extracellular ATP or of its derivatives (ADP, AMP and adenosine), probably due to its rapid metabolization (Falzoni et al., 1995; Joseph et al., 2003) by the ectonucleotidases CD39 and CD73, whose expression and activity were not modified by ABC. To detect instant slight ATP inputs in a specific membrane site responsible for cell signaling is challenging. Although we cannot completely discard the undetected ATP input, this limitation does not rule out the fact that ABC can interact directly with the P2X7R (docking model) facilitating its activation by ATP, as shown in the functional and calcium mobilization studies. In addition, while ATP induces a proinflammatory profile similar to that of ABC, some of the effects (leukocyte rolling velocity, rolling flux and expression of CD11b) were not prevented by specific blockade of P2X7R, therefore ruling out a role for this receptor. This was not entirely surprising, as P2X7R is considered a low affinity receptor that requires unusually high concentrations of ATP (>100 µmol/L) to become activated (Yan et al., 2010). In this context, the binding of three ATP molecules with an intact C-terminal tail (Karasawa and Kawate, 2016) triggers the opening of a non-selective pore that is permeable to high-molecular-weight solutes (Virginio et al., 1999; Mattig et al., 2003; Riedel et al., 2007; Riedel et al., 2007), mechanism previously associated with P2X7R cytotoxicity (Di Virgilio, 1995). While of obvious interest, these findings challenged our initial hypothesis of a relation between ATP and ABC, as the ATP concentrations that triggered pro-inflammatory actions in our setting were much lower (1 µmol/L) than those reported to induce direct activation of P2X7R.

Another important feature of P2X7R is its high degree of plasticity (Yan et al., 2010), attributed to the extended C-terminal domains of each monomer (Peverini et al., 2018). This opens up the prospect that its functioning involves allosteric regulatory mechanisms that act on extracellular or intracellular/transmembrane domains of the receptor subunits, altering their permeability, kinetics and agonist sensitivity (Surprenant et al., 1996). There is a growing list of positive and negative allosteric modulators of P2X7R (Martínez-Cuesta et al., 2020), including polymyxin B (Ferrari et al., 2004), clemastine (Norenberg et al., 2011), lysolipids (Michel and Fonfria, 2007), glycosaminoglycan (Moura et al., 2015), ginsenosides (Helliwell et al., 2015), other artificial pharmacological compounds (Michel et al., 2008), and, interestingly, nicotinamide adenine dinucleotide (NAD), a positive modulator with a purine structure like that of ABC (Hong et al., 2009). The discrepancies between the results of our binding and docking studies raise the possibility that ABC/ATP interplay is the result of an allosteric modulation of the receptor (Figure 1B4). Our docking calculations confirmed the possibility of two binding sites for ABC in P2X7R: site 1, defined as an ATP site; and site 2, occupied by the P2X7R antagonist A804598 and cataloged as an allosteric modulation site (Karasawa and Kawate, 2016). In our binding studies ABC could not shift radiolabeled A804598 from the P2X7R (unlike ATP), and in the docking analysis ATP was capable of binding to both sites (though it showed higher predicted affinity for site 1 while ABC would be binding only to site 2. Two observations during the docking predictions could explain the failure of ABC to displace the antagonist in these binding studies: 1) A804598 had a superior affinity to that of ABC for site 2 of P2X7R; and 2) both molecules (A804598 and ABC) could fit together into this site of the receptor, and consequently ABC could bind to it without displacing the antagonist. This is in line with recent evidence that has categorized A804598 as a non-competitive ATP-P2X7R antagonist since it has been proved to occupy a different site to that of ATP (Karasawa and Kawate, 2016).

This potential role of ABC, by which it boosts the actions of ATP, was confirmed from a functional point of view when low, ineffectual concentrations of ATP triggered a remarkable pro-inflammatory effect and calcium influx in the presence of low concentrations of ABC, actions that were mediated by P2X7R. Finally, Connexin43 channels were also implicated in the effects of low doses of ABC + ATP on leukocyte-endothelium interactions. These hemichannels allow the release of small molecules into the extracellular medium, and are responsible for ATP release in leukocytes (Vinken et al., 2010). Connexin43 channels are activated, among other stimuli, by increases in the intracellular concentration of calcium (Eltzschig et al., 2006). In addition, they are involved in inflammatory processes and purinergic receptor activation (Fortes et al., 2004; Locovei et al., 2006), and are located close to P2X7R in some vascular cells, such as macrophages (Fortes et al., 2004).

## Conclusion

**FIGURE 9:**
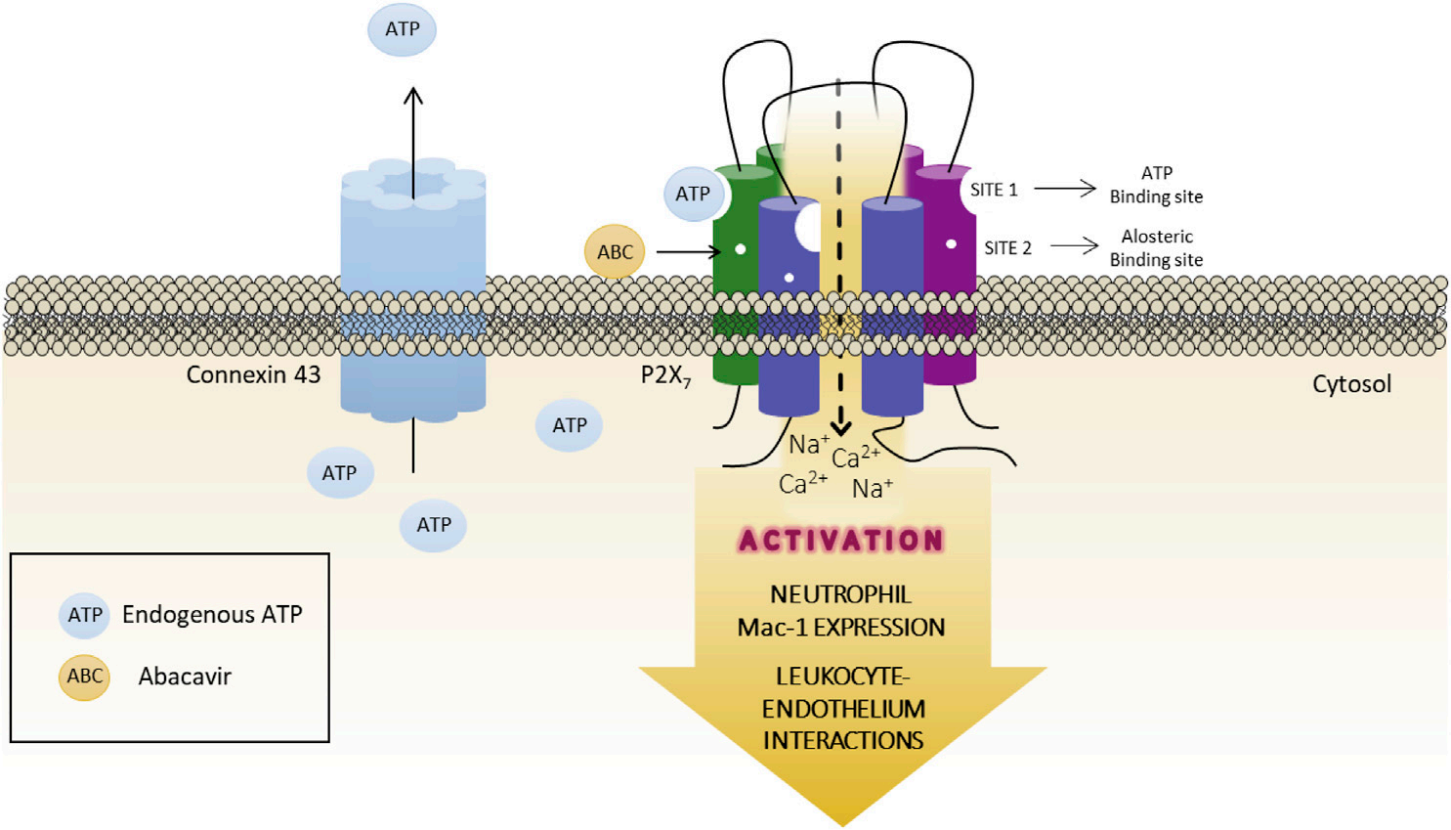
Graphical abstract. Proposed role for ABC acting as an allosteric modulator of the P2X7 receptor. ABC binds to a specific site of the receptor (site 2, allosteric binding site), but not to ATP-binding site (site 1), facilitating the endogenous ATP-P2X7R binding to site one and, thus, the activation of the receptor with low and physiological concentrations of ATP. This will lead to an increase in the expression of the adhesion molecule Mac-1 in PMN, which interacts with ICAM-1 on endothelial cells, causing the recruitment of leukocytes seen in the vascular inflammatory response induced by ABC. The release of ATP to the extracellular milieu through Connexin43 channels is also implicated in this response.

The present work suggests that the pro-inflammatory action of ABC involves an increased activation of ATP-P2X7R in human leukocytes, and that ABC binds to a specific site of the P2X7R (site 2) that is not the ATP-activating site (site 1). We hypothesize that ABC acts as a positive allosteric modulator that makes the receptor permeable to calcium; connexin channels are subsequently activated and ATP is released into the extracellular milieu, thus facilitating endogenous ATP-P2X7R binding and, in turn, their activation. In this scenario, ATP binds to site 1 of the receptor and, by inducing post-translation inflammatory mechanisms that could include inflammasome activation (NLRP3 and caspase -1 activation and IL-1β release) (Toksoy et al., 2017), increases expression of the adhesion molecule Mac-1 in PMN, which interact with ICAM-1 on endothelial cells, leading to the recruitment of leukocytes seen in the vascular inflammatory response induced by ABC (Figure 9). Whether a similar action could be elicited by other compounds and/or pathological conditions and be responsible for the various deleterious responses attributed to this widely expressed receptor is now open to discussion.

## Chemical Compounds

Abacavir sulfate (PubChem CID: 9843042), carbovir triphosphate (PubChem CID: 135499583), ATP (PubChem CID: 5957), A804598 (PubChem CID: 53325874), Suramin (PubChem CID: 5361), Apyrase (PubChem CID: 131750176), Carbenexolone sodium (PubChem CID: 636402), Probenecid (PubChem CID: 4911), Panx10 (PubChem CID: 44233736), Gap19 (PubChem CID: 91691126), and Polymyxin B sulfate (PubChem CID: 5702105).

## Data Availability

Inquiries regarding the original contributions presented in the study can be directed to the corresponding author.

## Abbreviations

ABC, Abacavir; NRTI, nucleoside reverse transcriptase inhibitor; HIV, human immunodeficiency virus, CBV-TP, carbovir triphosphate; ATP, adenosine triphosphate, P2X7R, purinergic ATP-P2X7 receptors; DPBS+, Dulbecco's PBS+; DPBS-, Dulbecco’s PBS-; HAS, human serum albumin; Bz-ATP, Benzoylbenzoyl-ATP; PE, phycoerythrin; FITC, fluorescein isothiocyanate; HUVECs, Human umbilical vein endothelial cells; PMN, human peripheral blood polymorphonuclear; CBX, Carbenexolone; Prob, Probenecid; FS, forward-angle light scatter; SS, side-angle light scatter; E-ATP, 1,N6-Etheno-adenosin-5′-triphosphate; E-ADP, 1,N6-Etheno-adenosin-5′-diphosphate; E-AMP, 1,N6-Etheno-adenosin-5′-monophosphate; E-ADO, 1,N6-Etheno-adenosin; LGA, Lamarckian genetic algorithm; panx1, pannexin; NAD, nicotinamide adenine dinucleotide; Pol, polymyxin B sulfate.
