# Supplementary material for: Abacavir Increases Purinergic P2X7 Receptor Activation by ATP: Does a Pro-inflammatory Synergism Underlie Its Cardiovascular Toxicity?
Source: Front Pharmacol. 2021 Mar 31;12:613449. doi: 10.3389/fphar.2021.613449 (PMC8045785; doi:10.3389/fphar.2021.613449)
Supplement: Supplementary file 1 [file datasheet1.docx]

Supplementary Material

# Supplementary Figures

## Supplementary Figure 1

**
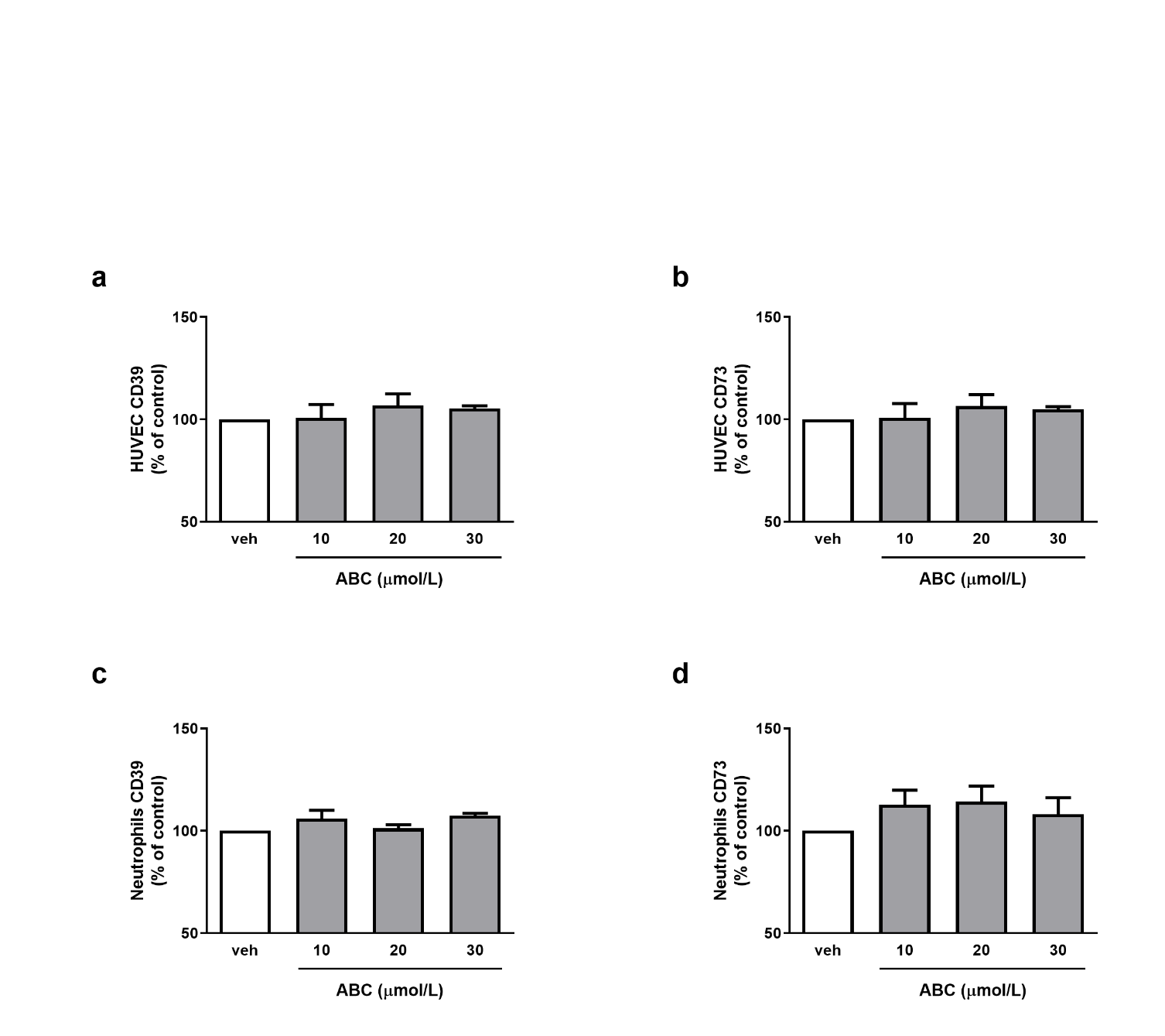
Supplementary Figure 1.** **Effects of abacavir on the expression of the enzyme proteins responsible for the degradation of nucleotides on endothelial cells and neutrophils**. HUVEC and PMN were incubated (4h) with Abacavir(ABC, 10-30 µmol/l) or sterile water (vehicle, veh) and the surface expression of CD39 (a and c) and CD73 (b and d) on HUVEC and PMN, respectively was quantified. Fluorescence values are expressed as percentage of mean of median fluorescence intensities of control cells (vehicle, 100%). Results are mean ± SEM, n≥4.

##
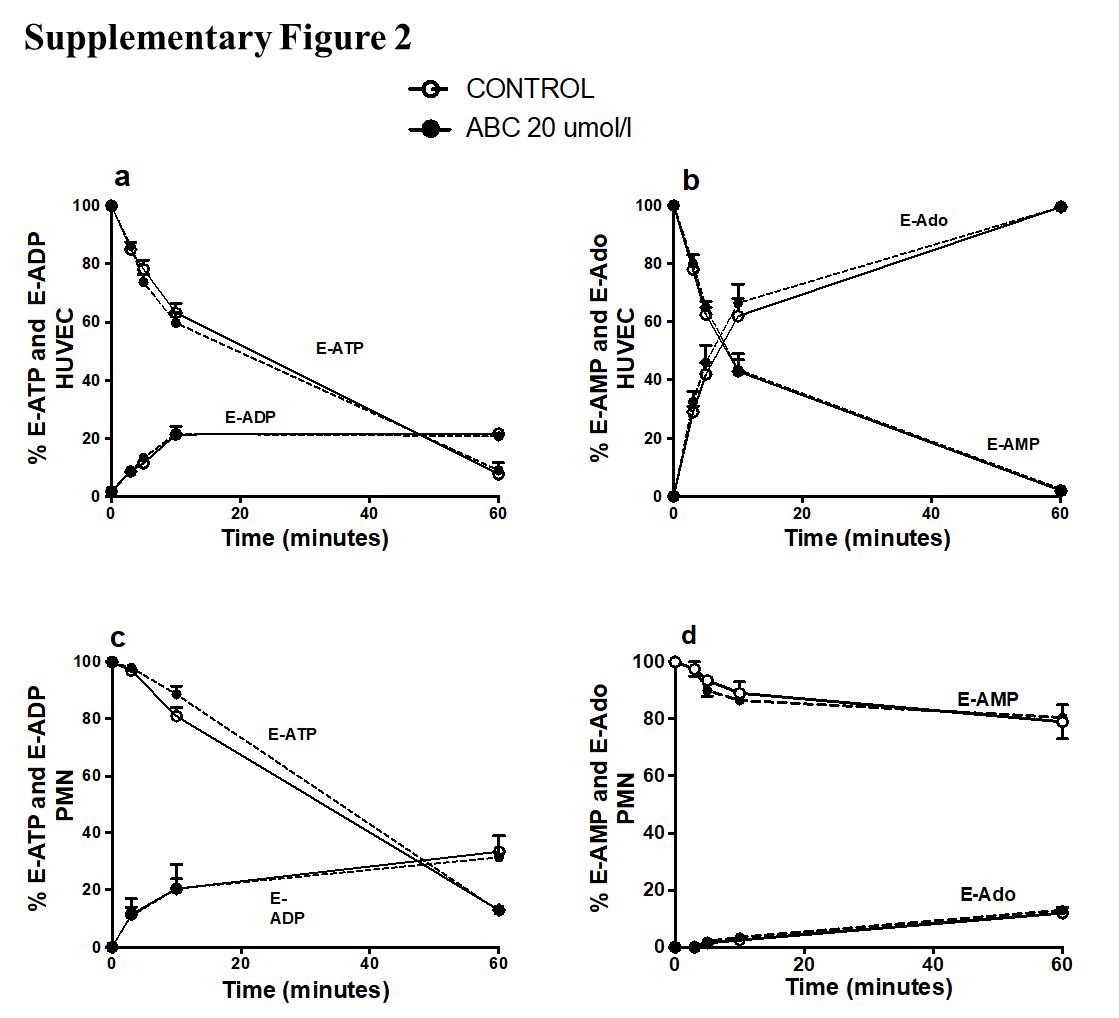
Supplementary Figure 2

## Supplementary Figure 2. Effects of abacavir on the enzymatic activity of the proteins responsible for the degradation of nucleotides on endothelial cells and neutrophils. HUVEC and PMN were incubated (4h) with Abacavir (ABC, 20 µmol/l, discontinuous line, filled circles) or vehicle (veh, continuous line, open circles). For CD73 activity, Etheno-ATP (E-ATP, 10 µmol/L) was added to both cell types and supernatants (0, 3, 10 and 60 min) were collected for quantification of the percentage of remnant E-ATP and the percentage of E-ADP which had been formed on HUVEC (a) and on PMN (c). For CD39 activity, Etheno-AMP (E-AMP, 10 µmol/L) was added to both cell types and supernatants (0, 3, 10 and 60 min) were collected for quantification of the percentage of remnant E-AMP and the percentage of E-Ado which had been formed on HUVEC (b) and on PMN (d). Results are mean ± SEM, n≥5.
